# Revisiting the Facility-Based Delivery Rate Formula in the Philippines for Better Local Health Governance and Services

**DOI:** 10.9745/GHSP-D-18-00256

**Published:** 2018-12-27

**Authors:** Fude Takayoshi, Sakiko Yamaguchi, Amelita M. Pangilinan, Makoto Tobe, Shogo Kanamori

**Affiliations:** a Koei Research & Consulting Inc., Tokyo, Japan.; b Department of Psychiatry, McGill University, Montreal, Quebec, Canada.; c Cordillera Administrative Regional Office, Department of Health, Baguio City, Benguet, Philippines.; d Japan International Cooperation Agency, Tokyo, Japan, and Research Center for Health Policy and Economics, Hitotsubashi Institute for Advanced Study, Tokyo, Japan.; e Department of Community and Global Health, Graduate School of Medicine, The University of Tokyo, Tokyo, Japan, and Department of Health, Manila, Philippines.

## Abstract

When calculating local facility-based delivery rates, the standard measure based on place of birth excludes residents' facility births outside the municipality. In contrast, counting the facility births of all residents—regardless of whether they take place within or outside their home municipality—provides a more accurate population- or residence-based measure of use of services for that catchment area. This residence-based measure offers local governments a better understanding of coverage gaps by taking into account place of residence rather than place of birth.

Data on health care-seeking behaviors are instrumental for identifying gaps in access to health services, an issue particularly important in the maternal and child health field as reflected in the United Nation's Sustainable Development Goals. However, lack of basic data, including data on health care services coverage at the community level, is an identified barrier to effective policymaking.^1^

The facility-based delivery (FBD) rate is an essential indicator because improving facility-based delivery with skilled birth attendants is an important strategy for lowering maternal mortality.^2^ Globally, the formula to compute the FBD rate from routine health information data is a simple one: the number of FBDs in a population divided by the total number of deliveries. However, at the community level, this formula might need to be redefined and clarified in terms of who should be counted in the numerator and denominator.

In the Philippines, the country's public health information system, the Field Health Services Information System, mandates the FBD rate to be occurrence-based, meaning that only deliveries that occur in a given place are counted:

$$\text{Occurrence-based FBD rate}=\frac{\text{Number of FBD cases that occurred in the municipality}}{\text{Number of deliveries that occurred in the municipality}}$$

As a basis for reporting, Barangay Health Workers (community health volunteers who provide health promotion and education) are tasked to track all pregnancy cases in their designated *barangays* (smallest administrative unit) through routine household visits, and nurses and midwives are in charge of filling out a register with individual information and place of delivery based on information obtained from the Barangay Health Workers and facility data. This register routinely captures 3 different scenarios of both facility- and home-based deliveries.

A. Residents' deliveries within municipality
B. Residents' deliveries outside municipality
C. Non-residents' deliveries within municipality

From this paper register, health workers report to the Field Health Services Information System by manually adding the numbers in Scenarios (A) and (C) for the denominator of the FBD rate. Likewise, FBD cases are extracted from this denominator and used as the numerator. Scenario (B), residents' deliveries outside the municipality, are not counted in either the denominator or the numerator.

In the Philippines' decentralized health system, local government units, such as provinces, municipalities, and *barangays*, have significant local autonomy and responsibility for managing and providing health care services.^3^ A problem arises when the occurrence-based FBD rate is officially employed as a proxy of the health care-seeking behaviors of pregnant women living in a municipality or *barangay*.

Take the Cordillera Administrative Region as a case in point. This ethnically diverse and mountainous region in the north-central part of Luzon has a population of 1,722,006 (as of 2015) and comprises 6 provinces and 1 chartered city.^4^ Besides cultural factors, geographic and economic barriers remain challenging problems to resolving unequal distribution of maternal and child health care, particularly access to health care facilities.^5^^,^^6^

In this area, pregnant women often cross the municipal boundaries to access health care services. Because of the mobility associated with their delivery practices, many health workers and mayors empirically know that the occurrence-based FBD rate is not suitable for local health planning. In response, a maternal and child health project, the Project for Cordillera-wide Strengthening of the Local Health System for Effective and Efficient Delivery of Maternal and Child Health Services (MCH-CAR) (2012–2017) supported by the Japan International Cooperation Agency, redefined the FBD rate formula from occurrence-based to residence-based by including in the numerator and the denominator all the deliveries of a municipality's residents, regardless of whether they occurred within or outside the home municipality:

$$\text{Residence-based FBDrate}=\frac{\text{Number of live birth FBD cases among residents of the municipality}}{\text{Number of live births among residents of the municipality}}$$

The occurrence-based FBD rate is not suitable for local health planning.

Note that the 2012 version of the Field Health Services Information System guideline indicates that both live births and stillbirths should be counted when computing the (occurrence-based) FBD rate.^4^ However, it is customary for field staff in practice to count only the live births. Therefore, to be consistent and comparative, the project team in collaboration with government counterparts counted only live births to compute both occurrence- and residence-based FBD rates.

The residence-based FBD rate takes into account all deliveries of a municipality's residents, regardless of whether they occurred within or outside the home municipality.

Figure 1 illustrates the potential difference obtained with the 2 formulas when considering 10 residents giving birth in 1 year, with 2 of the 10 delivering in a facility within the municipality, 6 delivering in a facility outside the municipality, and 2 delivering at home within the municipality. With the occurrence-based calculation, in which only the deliveries occurring within the municipality are counted, one obtains a 50% FBD rate (2 facility deliveries within the municipality/4 total deliveries). With the residence-based calculation, in which all residents' deliveries are counted, even those occurring outside the municipality, one obtains an 80% FBD rate (8 facility deliveries within and outside the municipality/10 total deliveries). The focus of attention and investment for a local government unit to address a 50% FBD rate is quite different than if the FBD rate were 80%.

**Figure:**
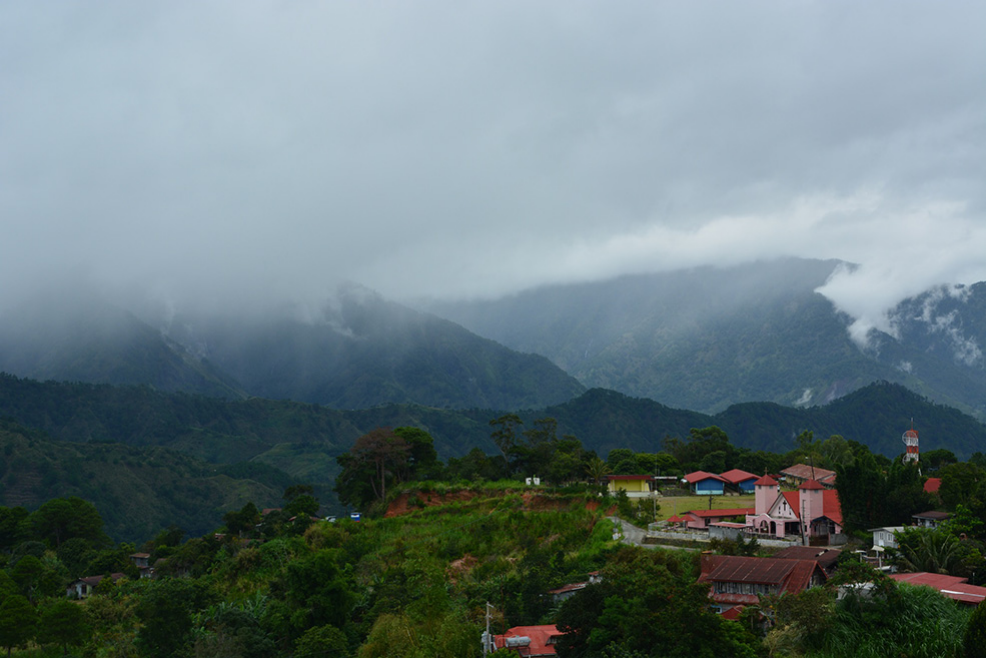
Typical mountainous scenery of Cordillera Administrative Region, Mankayan Municipality, Benguet Province, Philippines. ©2013/Amando Francisco Jr., MCH-CAR Project.

**FIGURE 1:**
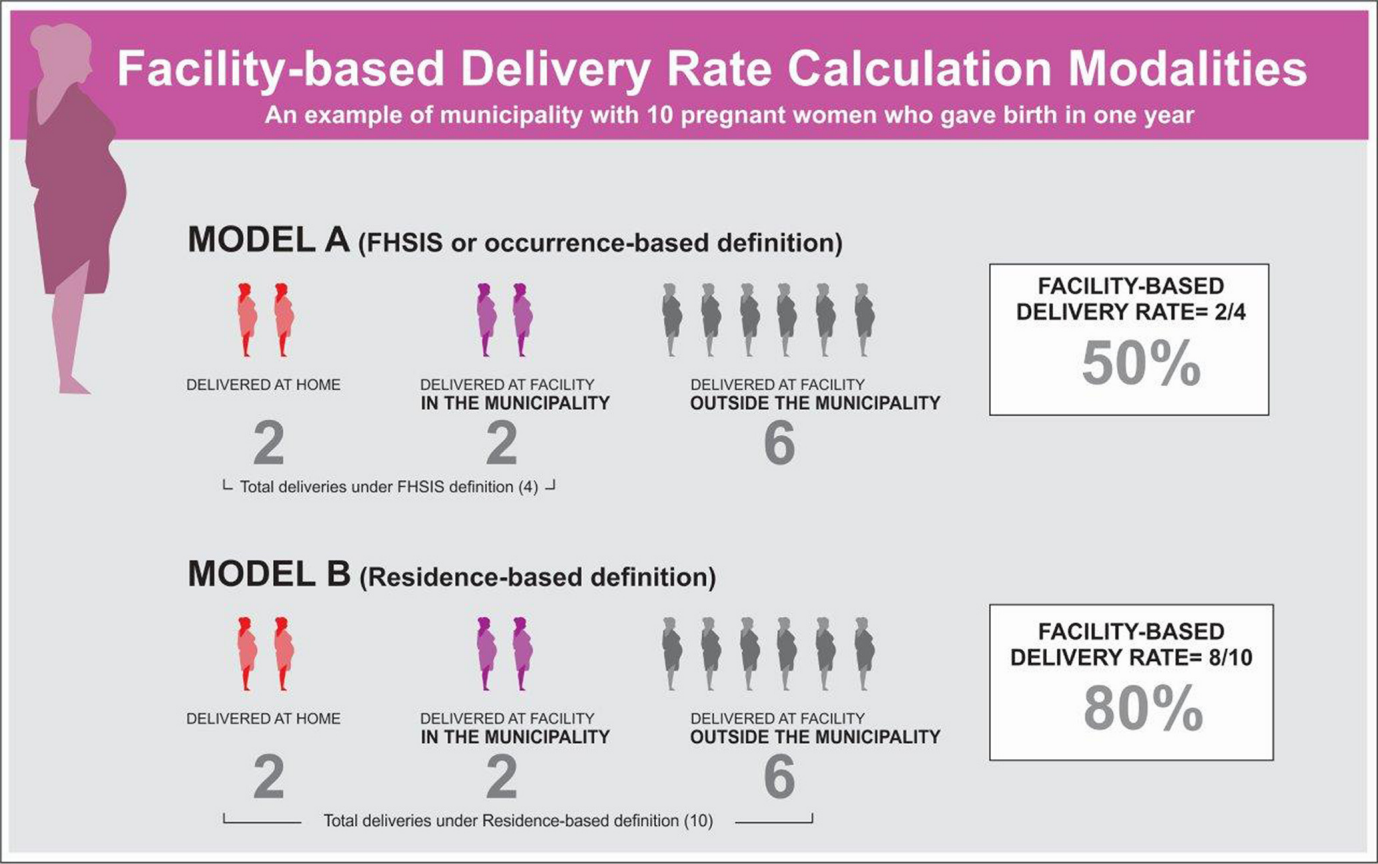
Comparison of Occurrence-Based With Residence-Based Computations of the Facility-Based Delivery Rate Abbreviation: FHSIS, Field Health Services Information System.

How feasible is it to apply this new equation? There is one favorable factor in the Cordillera Administrative Region: rural communities have close-knit social networks and are supportive of Barangay Health Workers doing rigorous pregnancy tracking. However, some health workers initially had difficulty distinguishing clearly between the 2 different definitions, and retraining them was required. To compute the residence-based FBD rate, they needed to pay closer attention to Scenario (B) while excluding Scenario (C).

Figure 2 shows the discrepancy between the 2 definitions in 13 municipalities for 2012 in 1 of the 6 provinces of the Cordillera Administrative Region, Benguet. The occurrence-based FBD rates did not provide a good population-based measure of service coverage in 7 of the municipalities (Itogon, Tuba, Sablan, Tublay, Bakun, Kabayan, and Kibungan). By inference, those low FBD rates could imply to policy makers—particularly those who do not understand how the occurrence-based FBD rate is calculated—that 62% (Tuba) to 100% (Sablan) of the deliveries were home-based, because the rates disregard the deliveries outside the residents' home municipality. Women in these 7 municipalities routinely travel to deliver in neighboring municipalities where hospitals are located.

**FIGURE 2:**
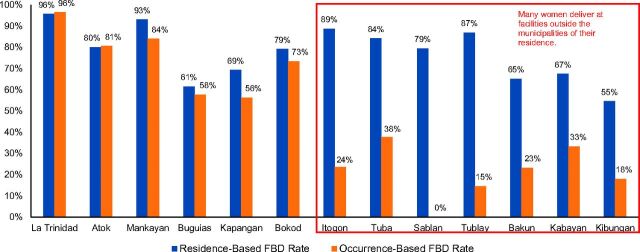
Facility-Based Delivery Rates of 13 Municipalities in Benguet Province, Philippines, 2012, by Computational Method Source: MCH-CAR project^7^ and Department of Health [Philippines].^8^

The extreme case is Sablan, which is adjacent to the chartered city. Because there were no birthing facilities in Sablan in 2012, the occurrence-based FBD rate was necessarily 0%. However, when factoring in the 93 pregnant women living in Sablan who delivered at facilities outside their home municipality and the 24 women who delivered at home, the residence-based FBD rate becomes 79%.

These results highlight that in this context low occurrence-based FBD rates in a municipality do not necessarily reflect low performance, or a high number of home-based births. Rather, the low FBD rates might point to the availability of hospitals and birthing centers outside the municipality. While building birthing facilities in all municipalities might seem an ideal health care solution, that is not necessarily the most effective way to distribute the limited health care resources of local government units.

Today, the Department of Health in the Cordillera Administrative Region continues to use residence-based data for its annual work and financial plan. With the redefined rate, local government units are able to capture their residents' delivery practices more precisely and tailor health policies to fit local needs more accurately. Further, using the redefined rate helps local officials identify geographical areas at relatively high risk by *barangay*. Understanding pregnant women's delivery practices and their mobility is crucial for effective health policymaking. This project experience highlights that revisiting nationally employed definitions of maternal and child health indicators may contribute to better local health governance and health care services.

## Notes

**Peer Reviewed**

**Funding:** None.
